# „Man muss aber auch selbst was tun“: Umgang mit dem Fibromyalgiesyndrom (FMS) – Perspektiven Betroffener und Behandelnder im Saarland

**DOI:** 10.1007/s00482-020-00504-7

**Published:** 2020-09-22

**Authors:** Marion Al-Qadi, Dagmar Renaud, Martha Meyer

**Affiliations:** Institut für Gesundheitsforschung und -technologie (igft), Hochschule für Technik und Wirtschaft des Saarlandes (htw saar), Goebenstraße 40, 66117 Saarbrücken, Deutschland

**Keywords:** Qualitative Forschung, Akzeptanz, Versorgung, Eigeninitiative, Netzwerke, Qualitative research, Acceptance, Health services, Self-initiative, Networks

## Abstract

**Hintergrund:**

Die Komplexität des Beschwerdebilds des Fibromyalgiesyndroms (FMS) stellt Betroffene wie auch Behandelnde vor besondere Herausforderungen. Die vorliegende Studie befasst sich mit den Fragen zum Umgang mit dem FMS aus Sicht Betroffener und Behandelnder und deren Einschätzung von Bedürfnissen bzw. Bedarfen in Bezug auf die Versorgung.

**Methodik:**

In einem qualitativen Studiendesign wurden die individuellen Sichtweisen von zehn FMS-Betroffenen und zehn Behandelnden untersucht. Die Personen wurden in einem „purposive sampling“ ausgewählt und in leitfadengestützten, problemzentrierten Interviews befragt. Die Auswertung erfolgte zusammenfassend inhaltsanalytisch.

**Ergebnisse:**

Betroffene und Behandelnde stellten eine problematische Verfügbarkeit und Zugänglichkeit von Versorgungsangeboten fest. Zudem fühlten Betroffene sich teilweise nicht ernst genommen von Behandelnden. Diese wiederum erlebten Vorbehalte gegenüber der psychosomatischen Komponente seitens der Betroffenen. Bedeutsam für einen positiven Umgang mit FMS scheint Eigeninitiative der Betroffenen zu sein.

**Schlussfolgerung:**

Selbsthilfegruppen und regional verfügbare Netzwerke stellen wichtige Unterstützungsmöglichkeiten dar, auch im Hinblick auf die psychosomatische Komponente. Durch Förderung interdisziplinärer Vernetzung kann eine verbesserte Koordination der Versorgungsleistungen bewirkt werden. Stärkung der Eigeninitiative Betroffener und Förderung der Arbeit der Selbsthilfegruppen helfen Betroffenen bei der Entwicklung individueller Bewältigungsstrategien.

## Abstract (en)

**Background:**

The complexity of the symptoms of fibromyalgia syndrome (FMS) poses a particular challenge for both those affected and those treating persons with the condition. The present study deals with the questions of coping with FMS from the perspective of both those affected and those treating them and their assessment of needs and requirements in terms of health services.

**Methods:**

In a qualitative study design, the individual views of 10 FMS patients and 10 healthcare providers were examined. The patients were selected by means of “purposive sampling” and questioned in guideline-based, problem-centred interviews. Evaluation was carried out by means of content analysis.

**Results:**

Both FMS patients and healthcare providers noted problems with availability and accessibility of health services. In addition, some patients felt that they were not taken seriously by their healthcare providers. Care providers in turn experienced reservations about the psychosomatic component on the part of patients. Self-initiative seems to be important for a positive approach to living with FMS.

**Conclusion:**

Self-help groups and regionally available networks represent important support possibilities, not least with regard to the psychosomatic component of FMS. Improved coordination of care services can be achieved by promoting interdisciplinary networking. Strengthening the self-initiative of patients and promoting the work of self-help groups can help those affected to develop individual coping strategies.

Das Fibromyalgiesyndrom (FMS) stellt ein komplexes Syndrom mit vielfältigen Beschwerden dar und kann für Betroffene eine gravierende Einschränkung ihrer Lebensqualität bedeuten. Bis zu Diagnosestellung und Finden individuell geeigneter Therapieformen müssen sie sich oftmals langwierigen Prozessen unterziehen. Im Umgang mit dem Syndrom sehen sich Betroffene wie Behandelnde^1^ mit besonderen Herausforderungen konfrontiert. In diesem Beitrag werden die Problemlagen zum Umgang mit dem FMS aus beiden Perspektiven vorgestellt.

Das Fibromyalgiesyndrom (FMS) tritt als komplexes Syndrom mit zahlreichen, häufig nicht trennscharf zu anderen chronischen Krankheitsbildern abgrenzbaren Beschwerden auf und ist gekennzeichnet durch die Hauptsymptome chronische Schmerzen in verschiedenen Körperregionen, Schlafstörungen bzw. nicht erholsamer Schlaf mit daraus resultierender Müdigkeit und Erschöpfungsneigung [2]. Die Prävalenzrate für Deutschland wird mit 2,1 % angegeben [13], international wird sie auf 2,7 % geschätzt [11]. Das weitgefächerte Beschwerdebild führt zu langwierigen Diagnostikprozessen, da verschiedene andere Erkrankungen systematisch ausgeschlossen werden müssen. Weitere Gründe ärztlicherseits könnten auch darin bestehen, dass die Möglichkeit des Vorliegens eines FMS nicht erkannt wird oder eine fehlende Akzeptanz des Syndroms als solches besteht [6]. Etliche Betroffene selbst warten zudem eine gewisse Zeit, bevor sie einen Arzt aufsuchen, was den Prozess zusätzlich beträchtlich verlängern kann. Als einer der Gründe wurde von den Betroffenen die Befürchtung genannt, mit ihren Beschwerden nicht ernst genommen zu werden [1]. Diese Faktoren können zu einem langen, häufig über mehrere Jahre dauernden Leidensweg führen [1, 3, 9]. So lag in einer deutschen multizentrischen Studie das Maximum bei 14 aufgesuchten Fachrichtungen und es konnte gezeigt werden, dass im Durchschnitt bereits 5,4 Jahre vor der Diagnosestellung eine chronische Schmerzgeneralisierung bei den Betroffenen stattgefunden hatte, wobei dem Zeitpunkt der Schmerzgeneralisierung mehr als 10 Jahre lang lokalisierte Schmerzen vorausgingen. Zudem müssen sich Betroffene oft zahlreichen Behandlungsversuchen unterziehen, bis sie eine individuell geeignete Therapie erhalten [9]. Aktuellere deutsche Studien zur beschriebenen Situation wurden nicht gefunden. Inwieweit sich die Situation seit Veröffentlichung der S3-Leitlinie möglicherweise geändert hat, kann daher nicht eingeschätzt werden.

Eine frühzeitige und korrekte Diagnosestellung kann dazu beitragen, unnötigen Belastungen der Betroffenen entgegenzuwirken. Die Autoren der S3-Leitlinie zum FMS gehen davon aus, dass das Erfahren einer Diagnose grundlegend für eine erfolgreiche Krankheitsbewältigung sein kann [2].

Im weiteren Verlauf müssen die Betroffenen lernen, mit den Beschwerden zurechtzukommen [4]. Während viele Studien den Erfolg von Behandlungen fokussieren, konnten für den deutschsprachigen Raum keine Studien zu individuellen Erfahrungen hinsichtlich der Versorgungssituation und des subjektiven Erlebens im Umgang mit dem FMS aus Sicht von Betroffenen und Behandelnden gefunden werden. Die Studie untersucht daher, wie Betroffene und Behandelnde den Umgang mit dem FMS aus ihrer Sicht einschätzen sowie welche Bedürfnisse bzw. Bedarfe es in Bezug auf die Versorgung gibt.

## Methodik

Da die Expertise der Behandelnden und die individuellen Erfahrungen aus der Lebenswelt der Betroffenen im Vordergrund standen, wurde ein qualitatives Design gewählt. Methodisch wurden problemzentrierte Interviews [12] eingesetzt. Die Leitfäden beinhalteten offene Fragen und wurden nach Prätests modifiziert (Tab. 1 und 2). Auf Basis eines „purposive sampling“ [10] wurden zehn vom FMS betroffene Personen interviewt. Die Kontaktaufnahme erfolgte über die saarländischen Selbsthilfegruppen, dabei wurde eine Beteiligung aus allen Landkreisen des Saarlandes angestrebt. Die Interviews wurden in sechs Fällen in deren Häuslichkeit und in vier Fällen in der Forschungseinrichtung geführt. Der Leitfaden enthielt Fragen zur Versorgungssituation, den Erfahrungen mit Behandelnden, zum Umgang mit der Erkrankung und der Alltagsbewältigung sowie zu Versorgungsbedürfnissen. Zunächst wurden mittels eines Kurzfragebogens Daten zur Beschreibung der Untersuchungspopulation erhoben. Die Interviewdauer betrug im Schnitt 38 min (13–79 min). Zudem wurden mit zehn Behandelnden verschiedener Fachrichtungen telefonische Interviews geführt. Die Auswahl erfolgte nach den Kriterien Fachrichtung und Landkreis. Der Zugang fand sowohl über das Fibromyalgie-Netzwerk Saar sowie über das Branchenbuch statt. Nach telefonischer Kontaktaufnahme wurde bei Bereitschaft zur Studienteilnahme ein Termin für ein Telefoninterview vereinbart. Behandelnde wurden ebenfalls zur Versorgungssituation, den Erfahrungen zum Umgang der Betroffenen mit dem FMS, der Akzeptanz des FMS sowie zu Versorgungsbedarfen befragt. Die durchschnittliche Interviewdauer betrug bei den Behandelnden 11 min (8–18 min). Alle Interviews wurden digital aufgezeichnet. Voraussetzung für die Teilnahme war die informierte Zustimmung gemäß den datenschutzrechtlichen Bestimmungen. Einverständniserklärungen erfolgten bei den Betroffenen schriftlich, bei den Behandelnden wurde jeweils eine mündliche Einverständniserklärung mit Zustimmung digital aufgezeichnet. Die Interviews wurden jeweils zeitnah vollständig wörtlich transkribiert [7] und qualitativ-inhaltsanalytisch ausgewertet [8]. Zur inhaltlichen Strukturierung der interessierenden Aspekte wurden zunächst deduktiv Kategorien gebildet, welche die im Leitfaden erfragten Themenkomplexe abbilden. Inhaltstragende Textstellen wurden den Kategorien zugeordnet und zusammenfassend dargestellt. Darüber hinaus wurden weitere Kategorien induktiv aus dem Material gebildet. Die gesamte Analyse wurde von zwei der Autoren (MA und DR) durchgeführt und die Ergebnisse verglichen. Es fanden sich weitgehende Übereinstimmungen, Abweichungen wurden diskutiert und im Konsens codiert.

**Table 1:** 

*Versorgung allgemein*	Wie schätzen Sie die Versorgungssituation von Fibromyalgie-Betroffenen im Saarland ein? (Zuständigkeit, Verfügbarkeit und Zugänglichkeit)
*Bedarfe*	Welche Bedarfe sehen Sie in Bezug auf die Versorgung?
	Wer übernimmt Ihrer Erfahrung nach die Koordination von Behandlungen?
	Wie sind Ihre Erfahrungen im Zusammenhang mit der interdisziplinären Zusammenarbeit mit Kollegen/Krankenkassen etc.?
	Verbesserungsvorschläge von Seiten der Behandler
*Akzeptanz*	Betroffene haben uns berichtet, dass ihre Beschwerden von manchen Behandlern nicht ausreichend ernst genommen werden. Kennen Sie dieses Phänomen und wie würden Sie es erklären?
*Erfahrungen im Umgang mit der Erkrankung*	Wie gehen Ihrer Erfahrung nach Betroffene mit der Erkrankung um?
*Abschließend*	Gibt es aus Ihrer Sicht noch etwas Wichtiges, was Sie gerne noch sagen möchten und bisher nicht angesprochen wurde?

**Table 2:** 

*Erfahrungen*	Wenn Sie nun an Ihr Fibromyalgiesyndrom denken: Wie hat das angefangen? Welche Erfahrungen haben Sie seither gemacht?
	Mit welchen Therapeuten/Behandlern/Institutionen hatten Sie im Verlauf Ihrer Erkrankung zu tun?
	Welche Erfahrung haben Sie dabei mit Ärzten und den anderen Behandlern gemacht?
	Wie sieht Ihre Therapie aus?
	Wie werden Entscheidungen in Bezug auf Behandlungen getroffen? Wie erleben Sie die Zusammenarbeit?
	Wie werden Angebote, Maßnahmen, Behandlungen organisiert? (Koordination)
	Selbsttherapie? Was hat geholfen, was nicht?
	Wovon oder von wem (Behandler/Institution) haben Sie bisher am meisten profitiert/wer oder was hat am meisten geholfen?
*Umgang mit Fibromyalgiesyndrom (FMS)*	Lassen Sie uns noch mal über Ihren Umgang mit dem Fibromyalgiesyndrom sprechen: Was hat sich für Sie verändert, seit Sie erkrankt sind? (Beruf, Freizeit, sozial, seelisch)
	Welche Unterstützung haben Sie bisher erfahren? (Selbsthilfegruppe)
	Wie erleben Sie den Umgang der anderen mit Ihrer Erkrankung? (Freunde, Familie, Kollegen, Behandler)
	Wie bewältigen Sie Ihre Erkrankung im Alltag?
*Bedürfnisse in Bezug auf Versorgung*	Was sollte sich ändern? (Wunsch an Politik/Gesundheitssystem)
*Abschließend*	Gibt es aus Ihrer Sicht noch etwas Wichtiges, was Sie gerne noch sagen möchten und bisher nicht angesprochen wurde?

## Ergebnisse

### Untersuchungspopulation Betroffene

Es wurden Interviews mit neun Frauen und einem Mann geführt, die zum Befragungszeitpunkt zwischen 51 und 62 Jahre alt waren. Es konnten Betroffene aus allen Landkreisen interviewt werden. Die Diagnosefindung dauerte im kürzesten Fall von Beginn der Beschwerden an etwa ein Jahr und erstreckte sich im Maximalfall über etwa 23 Jahre.

### Untersuchungspopulation Behandelnde

Die Teilnehmenden vertreten die Fachrichtungen Allgemeinmedizin, innere Medizin, Rheumatologie, Schmerzmedizin, Neurologie, Orthopädie und physikalische Rehabilitationsmedizin sowie die Fachgebiete Physiotherapie und Psychologie. Es konnten mit einer Ausnahme aus jedem Landkreis Studienteilnehmende rekrutiert werden. Es wurden jeweils fünf Frauen und Männer interviewt. Es konnten fünf Teilnehmende aus dem Fibromyalgie-Netzwerk Saar sowie fünf Teilnehmende ohne Netzwerkzugehörigkeit über das Branchenverzeichnis rekrutiert werden.

Im Folgenden werden die Ergebnisse gemäß den gebildeten *Haupt- und Unterkategorien *(Tab. 3) vorgestellt.

**Table 3:** 

*Versorgungssituation*	
*Erfahrungen Betroffener mit Behandelnden*	
*Akzeptanz des Fibromyalgiesyndroms (FMS) aus Sicht der Behandelnden*	Geringe Kenntnisse
	Zweifel an der Existenz
	Diffuses Erscheinungsbild
	Schwierige Diagnostik
*Umgang mit FMS*	Erlebte Unterstützung
	Eigeninitiative
	Akzeptanz der Einschränkung
*Versorgungsbedürfnisse*	
*Versorgungsbedarfe*	Koordinationsstruktur und Vernetzung
	Verordnungen
	Edukation, Empowerment, Fortbildungen

### Versorgungssituation

Sowohl Betroffene als auch Behandelnde berichteten von einem erschwerten Zugang (lange Wartezeiten auf Termine) insbesondere zu Psychotherapie, Schmerztherapie und Rheumatologie. Allerdings betonten einige Behandelnde, dass in manchen Fällen Psychotherapeuten bzw. die Psychosomatik nicht ausreichend einbezogen würden.

Betroffene berichteten von belastenden Auseinandersetzungen mit den Kostenträgern bzgl. Kostenübernahmen. Besonders hervorgehoben wurde die Schwierigkeit, ausreichend Verordnungen für Physiotherapie zu erhalten.

Insgesamt wurden die jeweiligen Therapieangebote als regional nur begrenzt verfügbar beschrieben, was einen nicht unerheblichen zeitlichen wie auch finanziellen Aufwand für die Betroffenen mit sich bringe (Fahrzeit und Fahrtkosten). Während für den städtischen Raum eine gute Verfügbarkeit und Zugang zu Versorgungsangeboten konstatiert wurden, sei es außerhalb der Städte deutlich schwieriger.

Alle Befragten sahen zu wenig spezialisierte Ansprechpartner bzw. Anlaufstellen. Laut der befragten Betroffenen mangele es an Behandelnden mit spezifischem Wissen über FMS.

Und der Orthopäde, der verschreibt einem dann mal Krankengymnastik, aber, wenn man da nicht zum richtigen Therapeuten kommt, bringt es einem auch nicht viel. Es ist halt einfach wichtig, dass man bei einen Therapeuten, Physiotherapeuten kommt, der sich auch mit Fibromyalgie auskennt (I7_63–66).

### Erfahrungen Betroffener mit Behandelnden

Ein zentrales Problem der Betroffenen bezieht sich auf ein Nicht-ernst-Nehmen ihrer Erkrankung und eine damit einhergehend erlebte abwertende Behandlung ihrer Person seitens einiger Behandelnden, die FMS nicht als Erkrankung anerkennen:

… was mir mal ein Arzt gesagt hat: Das ist die Krankheit, wenn man mal nicht arbeiten möchte (I9_169–170).

Des Weiteren klagten die Betroffenen über Desinteresse und mangelndes Verständnis in Verbindung mit Unwissenheit, sowohl seitens Behandelnder als auch medizinischen Assistenzpersonals. Daher raten einige Ärzte^2^ ihren Patienten, die Diagnose FMS zu tabuisieren und stattdessen von chronischem Schmerzsyndrom zu reden, das in der Fachwelt anerkannter zu sein scheint.

Einige Betroffene haben zudem die Erfahrung gemacht, dass mit feststehender Diagnose neu auftretende Symptome von den Behandelnden vorschnell dem FMS zugeschrieben werden.

… wir haben auch alles Mögliche, und das ist halt auch ein Phänomen, wo viele dann, … wenn die Diagnose gestellt wurde, gerne auf die Fibromyalgie abwälzen und keine Untersuchungen mehr machen (I10_334–336).

Als gute Erfahrungen wurden die Kompetenz, das persönliche Engagement und die Unterstützung einzelner Behandelnder hervorgehoben, insbesondere im Bereich der Allgemeinmedizin und der Schmerztherapie.

### Akzeptanz des FMS aus Sicht der Behandelnden

Mehrere befragte Behandelnde verwiesen auf die besondere Schwierigkeit der oft mangelnden Anerkennung bzw. Akzeptanz der Erkrankung seitens einiger Ärzte, infolgedessen sich Patienten häufig nicht ernst genommen fühlten.

Ich sag’ einfach mal, weil das von ärztlicher Seite her nach wie vor nicht so ernst genommen wird. Oder anerkannt wird. Oder erkannt wird (Int1_20–21).

Aus den Interviews lassen sich verschiedene Gründe der mangelnden Akzeptanz herleiten:

- **Geringe Kenntnisse: **Viele Behandelnde in der ambulanten Versorgung hätten geringe Kenntnisse zum Krankheitsbild, weil es noch zu wenig wissenschaftliche Erkenntnisse gebe und die Ursachen nach wie vor nicht bekannt seien.
- **Zweifel an der Existenz: **Einige Befragte schilderten Zweifel von Kollegen an der Existenz des FMS. Vereinzelte Befragte stehen dem FMS selbst skeptisch gegenüber, betrachten FMS als ein Konstrukt, für dessen Existenz es keinen Nachweis gebe.
  *„Also es geht nicht da drum, dass ich der Meinung bin, man schließt alles andere aus und wenn man nix gefunden hat, dann wird’s wohl die Fibromyalgie sein. Sondern ich bin der Meinung, dass es diese Erkrankung gar nicht gibt“ (Int9_39–43).*
- **Diffuses Erscheinungsbild: **Ein Befragter äußerte die Ansicht, für viele Ärzte sei es noch ein diffuses Krankheitsbild, da die von den Patienten geschilderten Beschwerden unklar seien und daher nicht ernst genommen würden.
  Weitere Befragte verwiesen auf große Vorbehalte von Kollegen aufgrund von Überlagerungen aus dem Bereich der somatoformen bzw. psychischen Störungen, der Schmerzsymptomatik und der Abgrenzung zu rheumatischen Erkrankungen.
  Manche Befragte vertraten die Auffassung, „*dass die Psychosomatik eine größere Rolle spielt“ (Int6_32–33)*, allerdings würden so manche Betroffene auf diese Richtung ablehnend reagieren.
  *„Deswegen geben die Patienten gerne an, dass sie nicht ernst genommen werden, aber das stimmt gar nicht, sondern es wird richtig gesehen“ (Int6_117–118).*
  Eine erfolgreiche Behandlung sei nicht allein durch die Behandlung von *„Schmerzen im Bewegungsapparat … auf orthopädischem Fachgebiet“*, sondern häufig auch *„durch die Behandlung eines psychischen Problems“ (Int9_46–49)* gekennzeichnet.
- **Schwierige Diagnostik**: Zu diesem Aspekt wurden zwei Schwerpunkte angesprochen.
  1. Fehlendes Diagnostikum, mit dem das FMS nachgewiesen werden könne.
    *„Bandscheibenvorfall kann ich greifen, eine Arthrose kann ich greifen, ich kann eine Hüft-TEP machen, aber die Fibromyalgie kann ich nicht messen, wie ich Zucker oder Blutdruck messen kann“ (Int2_58–60).*
  2. Eine weitere Schwierigkeit bestehe in dem langwierigen Diagnoseprozess aufgrund sehr langer Wartezeiten auf Termine bei Fachärzten und Psychotherapeuten (s. oben). Eine Diagnose sei aber wesentlich für die Verordnung von Behandlungen und regelmäßigen Therapien.

### Umgang mit FMS

- **Erlebte Unterstützung: **Die Betroffenen berichteten von unterschiedlichen Erfahrungen im Familien‑, Freundes- und Kollegenkreis, von Rücksichtnahme bis zu Unverständnis. Alle betonten hingegen den hohen Wert der Selbsthilfegruppen, wo sie sowohl Verständnis erfahren als auch von Informations- und Erfahrungsaustausch profitieren würden. Dazu wurde praktische Hilfe bei Formalitäten wie Rentenanträgen oder Widerspruchsverfahren positiv erlebt.
*„Was mir jetzt wirklich am meisten gebracht hat, ist die Selbsthilfegruppe“ (I3_175).*
  Auch die Behandelnden hoben die Tätigkeit der Selbsthilfegruppen für FMS-Betroffene sehr positiv hervor. Sie würden den Patienten als direkte Anlaufstelle und Informationsplattform dienen, Zugang zu Therapieangeboten schaffen und Rückhalt geben. Eine weitere Förderung und der Ausbau der Selbsthilfegruppen seien daher wichtig.
- **Eigeninitiative: **Alle Betroffenen äußerten, dass von ihnen ein hohes Maß an Eigeninitiative abverlangt würde, was ihre Versorgung im Rahmen des FMS betreffe. Die Befragten schilderten hierbei einerseits zunächst damit einhergehende zusätzliche Belastungen, andererseits schien gerade die aufgebrachte Eigeninitiative einigen Betroffenen langfristig die Erfahrung von Selbstwirksamkeit ermöglicht zu haben. Als hilfreich wurden individuelle Bewältigungsstrategien wie Entspannung, ausgewogene Ernährung und regelmäßige Bewegung empfunden. Dies hat sich offensichtlich positiv auf ihr eigenes Krankheitserleben und damit die individuelle Alltagsbewältigung mit FMS ausgewirkt.
*„Man muss aber auch selbst was tun“ (I9_180).*
  Auch Behandelnde sehen einen wesentlichen Aspekt für einen gelingenden Umgang in der Bereitschaft, die Lebensweise aktiv umzustellen, was Änderungsbereitschaft und Eigenverantwortung verlange. Hier gebe es Betroffene, die aktiv agierten und viel Eigeninitiative zeigten.
- **Akzeptanz der Einschränkung: **Als förderlich für ihre Alltagsbewältigung beschrieben Betroffene, die eigenen Grenzen der Belastbarkeit zu akzeptieren und diese nach außen klar zu kommunizieren.
  Ebenso beschrieben die meisten Behandelnden den Umgang der Betroffenen mit FMS als gut, sofern es gelungen sei, FMS als chronische Erkrankung zu akzeptieren, mit den damit verbundenen Einschränkungen im Alltag leben zu lernen und eigene Handlungsmöglichkeiten zu entwickeln.

### Versorgungsbedürfnisse (Betroffene)

Die Betroffenen wünschten sich Kostenübernahmen für individuell hilfreiche Therapien (z. B. osteopathische Behandlungen) sowie für frei verkäufliche Arzneimittel, die aber nicht zu offiziellen Kassenleistungen gehören (z. B. Enzympräparate). Darüber hinaus äußerte man Wünsche nach ganzheitlichen Therapieansätzen, speziellen Angeboten für FMS-Betroffene, zusätzlichen Verordnungen für Physiotherapie und entsprechenden flächendeckenden Angeboten. Ein weiterer wesentlicher Aspekt betrifft die Steigerung des Bekanntheitsgrads von FMS.

### Versorgungsbedarfe (Behandelnde)

- **Koordinationsstruktur und Vernetzung: **Kommunikation und Koordination, sowohl zwischen den Sektoren als auch zwischen einzelnen Berufsgruppen, wurden als wichtige Maßnahmen für die zukünftige Versorgung benannt, um den langwierigen, mit vielen Wartezeiten verbundenen Diagnoseprozess zu verbessern.
*„… es wäre halt ganz gut, denke ich, wenn es halt tatsächlich auch Koordinationsstellen gäbe oder dass man eine Koordinationsstruktur aufbaut“ (Int10_72–73).*
  Auch ein weiterer Ausbau und die Vernetzung der Selbsthilfegruppen wurden für wichtig erachtet, da die Selbsthilfegruppen große Unterstützung bieten könnten.
- **Verordnungen: **Verordnungen, vor allem für Physiotherapie, seien zu kurzfristig ausgelegt. In vielen Fällen sei eine ausgedehntere Behandlung wünschenswert. Aufgrund der Budgetierung sei es jedoch schwierig, trotz Notwendigkeit Rezepte zu erhalten oder zu verordnen.
- **Edukation, Empowerment, Fortbildungen: **In den Interviews wurde die Eigenverantwortlichkeit der Patienten für den erfolgreichen Verlauf von Therapien und Behandlungen betont und auf Aufklärung und Schulungen verwiesen. Die Anleitung zu selbstständigem Training, Bewegung und der Durchführung von Übungen trage wesentlich zum Therapieerfolg bei.
  Auch für Behandelnde werden Fortbildungsbedarfe zu FMS und seinen Therapiemöglichkeiten gesehen. Fortbildungen, Schulungen und Aufklärung der Behandelnden könnten zudem zur Akzeptanz beitragen.
  „*Das ist insgesamt eine schwierige Darstellung, aber sicherlich wird durch Fortbildung und durch, ja, auch Möglichkeiten der Therapie und Verlaufserfolge dann die Anerkennung größer, das könnte ich mir vorstellen“ (Int10_92; 97–98).*

## Diskussion

Die beschriebene Versorgungssituation unterscheidet sich hinsichtlich Zugänglichkeit und Verfügbarkeit kaum von anderen chronischen Krankheitsbildern mit entsprechendem Facharzt- oder Therapeutenbedarf. Das FMS weist allerdings aufgrund der schwierigen Diagnosestellung eine höhere Dichte der an Diagnostik und Behandlung beteiligten unterschiedlichen Facharztgruppen/Therapeuten auf, was die Situation für die Betroffenen besonders erschwert. Die S3-Leitlinie empfiehlt, dass Betroffene sich zur Behandlungskoordination an Ärzte wenden, die Kenntnisse und Erfahrungen in der Behandlung eines FMS haben sowie die Bereitschaft mitbringen, eine Lotsenfunktion zu übernehmen [2]. Auch die befragten Behandler in dieser Studie schlagen Vernetzung und koordinierte Zusammenarbeit der interdisziplinären Leistungserbringer zur Verbesserung dieser Situation vor. Für die Praxis würde das bedeuten, dass sich alle an der Versorgung und Behandlung Beteiligten entweder in Netzwerken organisieren oder sich bereits regional bestehenden Netzwerken anschließen und aktiv einbringen. In solchen Netzwerken könnten ganzheitliche bzw. spezielle Angebote entwickelt werden, was den Versorgungsbedürfnissen der befragten Betroffenen entgegenkäme.

Eine weitere, bereits beschriebene, Besonderheit betrifft die mangelnde Akzeptanz des FMS seitens einiger Ärzte [6]. Für die Betroffenen stellt eine mangelnde Akzeptanz ein zentrales Problem dar, da sie sich nicht ernst genommen, sondern abwertend behandelt fühlen.

Dieses Phänomen ist differenziert zu betrachten und beinhaltet zwei Aspekte. Ein Aspekt betrifft Annahmen der Behandelnden über die Existenz des FMS. Auch die in dieser Studie befragten Behandelnden äußerten auseinandergehende Meinungen. Die meisten Befragten waren von der Existenz des FMS überzeugt und hielten das Vorgehen im Sinne einer Ausschlussdiagnostik für einen notwendigen, wenn auch langwierigen Prozess. Andere Befragte verwiesen auf einen fehlenden Nachweis der Existenz des FMS. Aus Sicht der befragten Behandelnden ergibt sich diese Annahme aus bisher zu wenig Kenntnissen und Erkenntnissen zum FMS, dem diffusen Erscheinungsbild, einem fehlenden validen Diagnostikum und dem daraus resultierenden schwierigen Diagnostikprozess. Zur Steigerung der Akzeptanz schlagen die Behandelnden entsprechende Fortbildungsmaßnahmen vor.

Einen weiteren Aspekt der erlebten mangelnden Akzeptanz stellt die psychosomatische Komponente im Sinne des biopsychosozialen Modells chronischer Schmerzen dar. Diese Komponente wird von den Betroffenen und Behandelnden zum Teil kontrovers gesehen und kann entsprechend zu Konflikten führen. Behandelnde erleben manche Betroffene als nicht offen für diesen Aspekt. Umgekehrt fühlen diese Betroffenen sich auf psychische Probleme reduziert und es scheint für sie bedeutsam, eine Diagnose zu erhalten, die ihr Phänomen somatisch erklärt. Sie erleben demnach aus ihrer Sicht eine mangelnde Akzeptanz seitens der Behandler, die allerdings auf eigene Vorbehalte gegenüber der psychosomatischen Komponente zurückzuführen ist.

Diese negierende Haltung zum psychologischen Anteil sollte im Rahmen der Aufklärung über das Krankheitsbild besonders berücksichtigt werden. Hier scheint es wichtig, zunächst den Widerstand aufzulösen, um Akzeptanz auch dieser Dimension chronischer Schmerzen zu erreichen. Eine begleitende psychotherapeutische Unterstützung könnte so seitens der Betroffenen bereitwilliger in Anspruch genommen werden. In diesem Sinne könnte sie dem erfolgreichen Umgang mit dem FMS dienen sowie Empowerment ermöglichen.

Als besonders hilfreich könnte sich hier die Förderung der Eigeninitiative und Eigenverantwortung erweisen. Wie die Ergebnisse zeigen, hilft Eigeninitiative dem Finden individueller Bewältigungsstrategien und hilfreicher Therapiemaßnahmen. Dies führt zu einem Gefühl der Selbstwirksamkeit und unterstützt die Alltagsbewältigung. Grundsätzlich wird das Ausprobieren von Therapiemaßnahmen sowohl von Betroffenen als auch von Behandelnden als besonders wichtige Komponente im Umgang mit dem FMS betrachtet. Für die Betroffenen ist die Erkenntnis bedeutsam, selbst Handlungsmöglichkeiten zu haben und aktiv etwas tun zu können. Die Bedeutung des Selbstmanagements wird bereits im ersten deutschen Fibromyalgieverbraucherbericht zu Nutzen und Schäden von Therapieverfahren deutlich. Die von 1661 Teilnehmenden analysierten Daten zeigen, dass aktivitäts- und ruhebezogene Selbstmanagementstrategien (körperliche Aktivität bzw. Hinlegen und Entspannung) nicht nur am häufigsten angewendet werden, sondern ihnen zudem eine größere Wirksamkeit und Verträglichkeit als medikamentösen Therapieoptionen seitens der Betroffenen zugeschrieben werden [5]. Darin sollten die Betroffenen motiviert und ermächtigt werden (Empowerment).

Ein weiteres wichtiges Element neben der medizinischen und therapeutischen Versorgung ist die Arbeit der Selbsthilfegruppen. Bei diesen Anlaufstellen erhalten Betroffene nicht nur Hilfestellung bei Anträgen und Widerspruchsverfahren, sondern auch viele weitere hilfreiche Informationen. Besonders wichtig sind allerdings der Erfahrungsaustausch unter Gleichgesinnten und das gegenseitige Verständnis. Daher bieten die Selbsthilfegruppen eine große Unterstützung und deren Ausbau sollte auch aus Sicht der befragten Behandelnden weiter gefördert werden.

Den Vorschlägen der Befragten folgend wird eine zentrale Anlaufstelle sowohl für Behandelnde als auch Betroffene empfohlen. Sinnvollerweise sollte dies in enger Anbindung an bestehende Netzwerke erfolgen. Aufgaben dieser Fachstelle könnten bspw. sein:

- Öffentlichkeits- und Aufklärungsarbeit zur Steigerung des Bekanntheitsgrads des FMS
- Informationsarbeit zum FMS und seinen Behandlungsmöglichkeiten
- Fachkompetente Beratung für Behandelnde und Betroffene
- Förderung der Vernetzung
- Durchführung von Schulungs- und Fortbildungsmaßnahmen für Behandelnde
- Edukation (Aufklärung, Information, Schulung, Anleitung) und damit einhergehend Empowerment der Betroffenen
- Erstellung von Literatur- und Medienübersichten für Betroffene und Behandelnde
- Regelmäßige therapeutische Angebote, z. B. spezifische Bewegungsangebote (eventuell auch mit Forschungsvorhaben verbunden)
- Interessenvertretung auf gesundheitspolitischer Ebene

Ein komplexes Syndrom wie das FMS mit einer hohen Dichte verschiedener Leistungserbringer braucht Koordination und Kooperation auf Fall- sowie auf Systemebene. Eine Herausforderung für die Zukunft wird sein, die interprofessionelle Zusammenarbeit weiter zu entwickeln, um eine qualitativ hochwertige Versorgung zu gewährleisten. Zu bedenken ist, dass Koordination und Kooperation in interprofessioneller Zusammenarbeit Zeit in Anspruch nehmen und von den Akteuren entsprechende Ressourcen erfordern. Leistungen für Kommunikation, Kooperation und Koordination sollten für alle beteiligten Leistungserbringer entsprechend vergütet werden.

## Limitationen

Folgende Aspekte könnten die Aussagekraft der Ergebnisse einschränken:

- Studie ist auf Region Saarland beschränkt
- Positive Selektion der Interviewteilnehmer aus den Selbsthilfegruppen, die mit ihrer Teilnahme bereits ein gewisses Maß an Eigeninitiative erwiesen
- Es konnten nicht aus allen Landkreisen Behandelnde für ein Interview gewonnen werden

## Fazit für die Praxis

Für die Betroffenen stellt es ein gravierendes Problem dar, wenn sie den Eindruck haben, das Fibromyalgiesyndrom (FMS) werde seitens der Behandler nicht ernst genommen. Allerdings können manche Betroffene die psychosomatische Komponente schwer annehmen, was zu dem Gefühl mangelnder Akzeptanz führen kann. Hier sollten zunächst Vorbehalte gegenüber diesem Aspekt aufgelöst werden, damit eine begleitende psychotherapeutische Unterstützung in Anspruch genommen wird. Als besonders hilfreich haben sich zudem in Eigeninitiative gefundene Selbstmanagementstrategien wie regelmäßige Bewegung und Entspannung erwiesen. Darüber hinaus stellen Selbsthilfegruppen sowie regional verfügbare Netzwerke wertvolle Unterstützungsmöglichkeiten dar, gerade auch im Hinblick auf die psychologischen Anteile. Eine Vernetzung auch unter den Behandelnden befördert die interdisziplinäre Zusammenarbeit und dient der Weiterentwicklung der Versorgungsangebote.

## Footnotes

1 Unter dem Begriff „Behandelnde“ werden in diesem Beitrag alle diejenigen ärztlichen und therapeutischen Berufsgruppen zusammengefasst, die an der Behandlung der Fibromyalgiesyndrombetroffenen beteiligt sind.

2 Aus Gründen der besseren Lesbarkeit wird an einigen Stellen das generische Maskulinum verwendet. Dies schließt stets die weibliche und männliche Form jeweils gleichermaßen ein.
